# Slit3 inhibits Robo3-induced invasion of synovial fibroblasts in rheumatoid arthritis

**DOI:** 10.1186/ar2955

**Published:** 2010-03-18

**Authors:** Alexandra E Denk, Simone Kaufmann, Klaus Stark, Jörg Schedel, Torsten Lowin, Thomas Schubert, Anja K Bosserhoff

**Affiliations:** 1 Institute of Pathology, University of Regensburg, Franz-Josef-Strauss-Allee 11, 93053 Regensburg, Germany; 2 Department of Internal Medicine II, University of Regensburg, Franz-Josef-Strauss-Allee 11, 93053 Regensburg, Germany; 3 Department of Internal Medicine I, University of Regensburg, Franz-Josef-Strauss-Allee 11, 93053 Regensburg, Germany

## Abstract

**Introduction:**

The repellent factor family of Slit molecules has been described to have repulsive function in the developing nervous system on growing axons expressing the Robo receptors. However, until today no data are available on whether these repellent factors are involved in the regulation of synovial fibroblast (SF) activity in rheumatoid arthritis (RA).

**Methods:**

mRNA expression in primary synovial fibroblasts was quantified by quantitative reverse transcription PCR and protein expression was measured by fluorescence activated cell sorting (FACS) analysis. Different functional assays were performed with rheumatoid arthritis synovial fibroblasts (RASF): proliferation, migration and a novel *in-vitro *cartilage destruction assay.

**Results:**

First, we found increased expression of Robo3 expression in RASF compared to normal SF. Interestingly, analysis of data from a recently published genome-wide association study suggests a contribution of *ROBO3 *gene polymorphisms to susceptibility of RA. Functional assays performed with RASF revealed induction of migration and cartilage destruction by Robo3 and increased matrix metalloproteinase (MMP)1 and MMP3 expression. Treatment of RASF in early passages with Slit3 led to inhibition of migration whereas RASF in later passages, having reduced Robo3 expression in cell culture, were not inhibited by Slit3 treatment. Here, reduction of Robo3 expression from passage 3 to 10 might reflect an important step in losing repulsive activity of Slit3.

**Conclusions:**

Taken together, our data showed that deregulation of the Robo3 receptor in synovial fibroblasts in RA correlates with aggressiveness of the fibroblasts. Slit3 reduces the migratory activity of synovial cells from patients with RA, potentially by repulsion of the cells in analogy to the neuronal system. Further studies will be necessary to prove Slit activity *in vivo*.

## Introduction

A strict separation of compartments is essential to ensure the correct mechanical function of the joint. Alterations at the joint compartment boundaries are frequently found in inflammatory or degenerative joint diseases such as rheumatoid arthritis (RA). The destruction of articular structures of the joint, such as cartilage and bone, through synovial fibroblasts (SF) is a crucial event especially in RA [1,2].

The chronic autoimmune disease RA is of unknown origin but finally leads to joint destruction [1]. The so-called 'tumour-like' or 'activated' SF are localized in the hyperplastic synovium of patients with RA. Supported by adhesion molecules, these rheumatoid arthritis synovial fibroblasts (RASF) attach to cartilage, where matrix-degrading enzymes released by RASF finally cause the destruction of the joint [3].

As loss of integrity of compartment borders in the joint between cartilage and SF is a key event in RA, we were interested in analyzing molecular cues, such as the repellent factors, that might be deregulated in RA patients and thereby contribute to destruction of joint borders.

Repellent factors of the Roundabout (Robo)- and Slit-family are primarily known to be involved in regulating cell-cell interactions and cell-matrix interactions of migrating cells during embryonic development [4] and by mediating axon guidance through attraction or repulsion of growth cones [5-7]. Over the past decade, the Robo-/Slit-system has also been described to mediate cell adhesion of fibroblasts [8] and to induce tumor angiogenesis [6].

There are four human Robo transmembrane receptors (Robo1, Robo2, Robo3, and Robo4) that share fibronectin type III and immunoglobulin (Ig)-like domains, but vary in their cytoplasmatic domains. The ligands for the Robo receptors are the secreted Slit molecules (Slit1, Slit2 and Slit3) consisting of four leucine-rich repeat domains (D1 to D4), seven to nine epidermal growth factor-like domains, a laminin G domain, and a C-terminal cysteine-rich domain [5]. Slit binding to Robo receptors is mediated by the second of the four highly conserved leucine-rich repeat domains of the ligand Slit and the first extracellular Ig domain of the Robo receptor [9-11].

In our study, we first analyzed the expression of Robo1, Robo2, Robo3, Slit1, Slit2, and Slit3 in the SF of patients with RA and continued to evaluate the influence of Robo3 expression and effects of Slit3 on RASF as a potential therapeutic tool.

## Materials and methods

### Primary fibroblasts

Synovial tissue samples were obtained from synovectomy and arthroplastic surgery from patients with RA (RASF) or from trauma surgery patients (normal SF) after informed consent and approval of the local ethics committee. All RA patients fulfilled the American College of Rheumatology 1987 criteria for the diagnosis of RA. In RA patients, material was sampled from the wrist or proximal interphalangeal joints with the joints exhibiting florid synovitis and/or arthritic destructions. Normal SF deriving from normal synovial tissue was taken from knee joints of two patients within two to four hours after knee injury. Both patients underwent surgery because of displaced tibial plateau fractures. Synovial tissue was minced mechanically, washed extensively in sterile PBS and digested with 150 mg/ml Dispase II (Boehringer Mannheim, Mannheim, Germany) for one hour at 37°C under continuous agitation. The resulting cell suspension was seeded into tissue culture dishes and cultured in DMEM (Gibco Life Technologies, Basel, Switzerland) containing 10% FCS and 100 U/ml penicillin per 100 μg/ml streptomycin in a humidified atmosphere at 37°C followed by the addition of 5% carbon dioxide. In different passages, adhering fibroblasts were washed, trypsinized, and used for RNA isolation or in the assays.

Normal dermal fibroblasts were obtained and used as described previously [12].

The number of donors used in each experiment is given in each figure legend.

### Transient transfection

Primary fibroblasts were transfected using the Amaxa Nucleofector System (Amaxa, GmbH, Cologne, Germany) with the Amaxa Normal Human Dermal Fibroblast Nucleofector^® ^Kit according to the manufacturer's instructions (program: U23) [13]. The transfection efficiency was about 30%, toxicity was 20%.

For analysing Robo3 effects, synovial cells in late passages (P5 and P6) or dermal fibroblasts were transiently transfected with mouse Robo3 expression construct, which was a gift from Shyng-Shiou Yuan [14]. Vector pCMX [13] was used as control. Transfection efficiency was quantified by quantitative real-time (RT)-PCR.

### Genetic association study

Genome-wide data from a recently published screen for polymorphisms associated with RA were employed to survey the complete *ROBO3 *gene region *in silico *[15]. All successfully genotyped SNPs on the Affymetrix GeneChip^® ^Human Mapping 500K Array Set (Santa clara, CA, USA) on chromosome 11 in the *ROBO3 *region with approximately 40 kb on either side of the gene (position 124,200,001 and 124,300,000; human genome build 18) were included. Wellcome Trust Case Control Consortium (WTCCC) data were accessed on by 26 June 2007 [15,16]. Data of the HapMap phase II release 22 data were used to assess linkage disequilibrium patterns of the *ROBO3 *gene region [17].

### RNA isolation and reverse transcription

Total cellular RNA was isolated from cultured cells using the RNeasy kit (QIAGEN, Hilden, Germany) and cDNAs were generated by reverse transcriptase reaction as described previously [18].

### Quantitative RT-PCR

Quantitative RT-PCR was performed on a LightCycler (Roche, Mannheim, Germany) using 10 μl N', N'-dimethyl-N-[4-[(E)-(3-methyl-1,3-benzothiazol-2-ylidene)methyl]-1-phenylquinolin-1-ium-2-yl]-N-propylpropane-1,3-diamine (SYBR) MIX (TaKaRa, Shiga, Japan), 0.5 μl (20 μM) of forward and reverse primers and 1 or 2 μl cDNA template in a total of 20 μl. cDNA fragments of Robo1, Robo2, Robo3, Slit1, Slit2, and Slit3 in SF in early passages (P3 and P4) were amplified according to the following PCR program: 30 seconds at 95°C (initial denaturation); 20°C/second temperature transition rate up to 95°C for 5 seconds, 10 seconds at 55°C (Robo1, Robo2, Slit2)/56°C (Slit1, Slit3)/58°C (Robo3), 15 seconds at 72°C, 10 seconds at 82°C (Robo1, Robo2, Slit2)/86°C (Slit1, Slit3)/85°C (Robo3) acquisition mode single, repeated for 40 times (amplification).

Matrix metalloproteinase (MMP)-1 and MMP-3 quantitative RT-PCR was performed 48 hours after transfection at 95°C for 5 seconds, 3 seconds at 68°C (MMP-1) or 62°C (MMP-3), 10 seconds at 72°C and 8 seconds at 82°C (MMP-1) or 82°C (MMP-3) acquisition mode single. Annealing temperature was optimized for each primer set and the PCR reaction was evaluated by melting curve analysis following the manufacturer's instructions and checked by electrophoresis. Beta-actin mRNA was amplified to ensure cDNA integrity and to normalize expression. Each quantitative PCR was performed at least in duplicate for two sets of RNA preparations.

The following primers were used: Robo1 (for: AGG AAG AAG ACG AAG CCG AC, rev: CGA AGA ACT AAC ACT GGA GCG), Robo2 (for: GAG ACC TCA CAA TCA CCA ACA TTC AAC, rev: CAG TAA CGC TGT ACC ATC CAC TGC), Robo3 (for: GCGCTTCTCAGTGTCTCCAAG, rev: TGGTCCCTGGAGGATGACA), Slit1 (for: ACT CGC TGG TCC TCT ATG GAA, rev: CGC AAA TGA AAG GGT TCT GGG), Slit2 (for: TGC CTT TGC CCA CCT GAG TA, rev: TGT CGC AGT GTT CAC CTA CG), Slit3 (for: TGA TGG CAA CGA GGA GAG TA, rev: ACG GCT GTT AGG TGG TTT CC) MMP1 (for: TGG ACC AAG GTC TCT GAG GGT CAA, rev: GGA TGC CAT CAA TGT CAT CCT GA) and MMP3 (for: GGC ACA ATA TGG GCA CTT TAA ATG AAG C, rev: GTC TAC ACA GAT ACA GTC ACT TGT CTG).

### Cell proliferation assay

Proliferation was measured using the Cell Proliferation Kit II (Roche,, Mannheim, Germany) according to the supplier's instructions. It is based on a colorimetric (XTT) measured non-radioactive quantification of cell proliferation performed for four days. RASF in passage 3 and 4 were treated with recombinant mouse Slit3 (R&D Systems, Minneapolis, MN, USA) in a concentration of 0.1 μg/ml. Experiments were repeated at least twice.

### Migration assay

Migration assay were performed using Boyden Chambers containing polycarbonate filters with 8 μm pore size (Costar, Bodenheim, Germany), as described previously [18]. Briefly, filters were coated with gelatin. The lower compartment was filled with fibroblast-conditioned medium, used as a chemo-attractant. Synovial cells in early passages (P3 and P4) and in late passages (P5 and P6; as indicated) were harvested with trypsin incubation for two minutes, resuspended in DMEM without FCS at a density of 3 × 10^4 ^cells/ml and injected in the upper compartment of the chamber on the filter. After incubation at 37°C for four hours, all cells attached to the upper surface of the membrane were removed. Cells adhering to the lower surface were fixed, stained and counted. Recombinant mouse Slit3 (R&D Systems, Minneapolis, MN, USA) was added either to the upper or the lower chamber of the system in a concentration of 0.1 μg/ml. All experiments were performed in triplicates and repeated at least twice.

### Cartilage destruction assay

Cartilage was obtained from donors after informed consent and approval of the local ethics committee. Normal cartilage with no significant softening or surface fibrillation was obtained at autopsy within 48 h after death. Cartilage was kept at -80°C before use. SF (50,000 cells per attempt) in early passages (P3 and P4) and synovial cells in late passages (P5 and P6) or dermal fibroblasts with Robo3 transfection (as indicated) were incubated with pieces of cartilage (8 pieces of 2 mm^3 ^(50 mg) per attempt) in 400 μl of medium for 10 days. After 10 days, the supernatant was collected and amounts of glycosaminoglycans (GAG) were measured using the sGAG quantitative kit (Euro-Diagnostica, Malmo, Sweden). Each attempt was measured in parallel and repeated twice with different donors of SF.

### FACS analysis

To analyse Robo3 expression on RASF, cells in early passages (P3 andP4) were detached from flasks using 5 mM EDTA in PBS. Cells were resuspended in PBS and 2.5 × 10^5 ^cells per approach were fixed in 100% Methanol for 10 minutes, twice washed with PBS and permeabilized with 0.1% Tween/PBS. Cells were washed twice with PBS and incubated with anti-Robo3 at room temperature for one hour (1:50; Everest Biotech, Oxfordshire, UK) in 1% BSA/PBS. After three washing steps with PBS, the cells were stained with the Cy™5-conjugated anti-goat secondary antibody (1:100, Jackson ImmunoResearch, Suffolk, UK) in 1% BSA/PBS for 30 minutes. After three washing steps the samples were resuspended in 250 μl PBS. All steps were performed at 4°C. FACS data were analyzed using the BD FACSDiva software (Becton Dickinson, Heidelberg, Germany) and WinMDI. RASF from four donors were analyzed.

### ELISA

Pro- and active form of MMP1 (n = 3) and MMP3 (n = 4) were analysed with RayBio Human MMP-1 and MMP-3 ELISA Kit (Norcross, GA, USA). Supernatant of mRobo3 and control transfected synovial cells used in the GAG assay was diluted 1:50 in one times assay diluent and subsequent analysed according to the manufacturer's instructions.

### Statistical analysis

Calculations were performed using the GraphPad Prism software (GraphPad software Inc, San Diego, CA, USA). All results are expressed as mean +/- standard deviation (range) or in percentage. Error bars represent standard deviation. Comparison between groups was performed using the Student's t-test.

## Results

### Robo and Slit expression in RASF compared with normal SF

**Figure 1:**
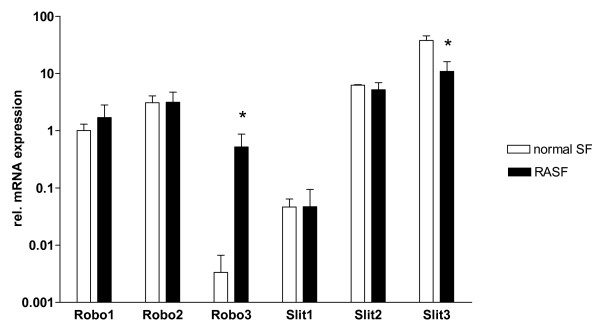
**Robo and Slit expression in RASF**. The amount of Robo1, Robo2, Robo3, Slit1, Slit2 and Slit3 mRNA expression in rheumatoid arthritis synovial fibroblasts (RASF; n = 5) compared with normal SF (n = 2) in early passages (P3 and P4) was quantified by real-time (RT)-PCR (logarithmic scaling). Robo1 and Robo2 are both expressed in normal SF and RASF, whereas Robo3 is much stronger expressed in RASF compared with normal SF. Slit1, Slit2, and Slit3 are all expressed in normal SF and RASF, with significant reduction of Slit3 expression in RASF (* *P *< 0.05).

First, we characterized the expression pattern of the receptors Robo1, Robo2, Robo3, and their ligands Slit1, Slit2, and Slit3 in RASF compared with normal SF in early passages (P3 and P4; Figure 1). Robo1 and Robo2 were almost equally expressed in normal SF and RASF, whereas Robo3 mRNA was strongly enhanced in RASF compared with SF of healthy donors.

Slit1 mRNA levels were lower than Slit2 or Slit3 mRNA levels; however, only differences in expression of Slit3 were found comparing RASF and normal SF.

### Genetic association of Robo3 and RA

Next, the potential genetic association of Robo3 and RA was analysed. The WTCCC genome-wide association study on RA [15] surveyed 15 SNPs within the *ROBO3 *gene region on chromosome 11 (position 124,200,001 and 124,300,000; human genome build 18). Four SNPs showed nominal association with susceptibility to RA in an additive model (Table 1). The two markers with the strongest association signal (rs11604758 and rs3923890) are in weak linkage disequilibrium (r^2 ^= 0.15).

**Table 1:** Results from WTCCC RA GWAS in *ROBO3 *gene region

SNP	Position ^a^	*P *value ^b^	Function
rs653403	124,201,501	0.329954	5' intergenic *ROBO3*
rs610104	124,213,908	0.217045	5' intergenic *ROBO3*
rs10790711	124,219,899	**0.0341778**	5' intergenic *ROBO3*
rs733601	124,220,090	0.762976	5' intergenic *ROBO3*
rs1940177	124,223,175	0.859324	5' intergenic *ROBO3*
rs11604758	124,226,213	**0.00078241**	5' intergenic *ROBO3*
rs11219814	124,233,333	0.184905	5' intergenic *ROBO3*
rs4936957	124,245,198	**0.0184723**	Intron 4*ROBO3*
rs3923890	124,245,620	**0.00875808**	Intron 5*ROBO3*
rs10790714	124,255,238	0.0629092	Intron 26 *ROBO3*
rs4326810	124,255,347	0.163546	Intron 26 *ROBO3*
rs12823	124,259,821	0.0722958	3' UTR *ROBO4*
rs11219831	124,267,979	0.421456	Intron 10 *ROBO4*
rs11219832	124,272,500	0.703442	Intron 1 *ROBO4*
rs4077566	124,276,481	0.0658315	5' intergenic *ROBO4*

^a ^on chromosome 11 (hg18).

^b ^additive model.

Nominal significant results are displayed in bold.

GWAS, genome-wide association study; RA, rheumatoid arthritis; SNP, single nucleotide protocol; WTCCC, Wellcome Trust Case Control Consortium.

### Functional role of Robo3 in RA

**Figure 2:**
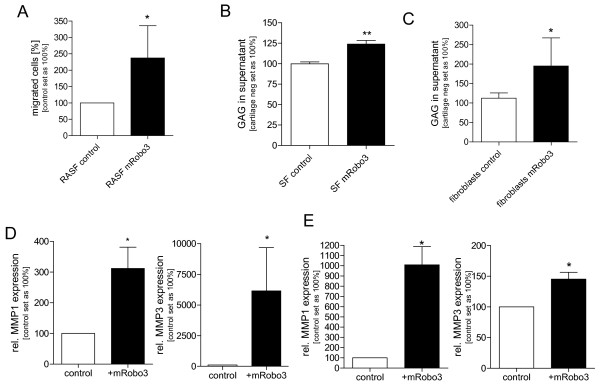
**Effect of Robo3 on RASF migration and aggressiveness**. **(a) **Transfection of RASF cells (late passage) with an expression plasmid for Robo3 increased migration of rheumatoid arthritis synovial fibroblasts (RASF; n = 4) in the Boyden Chamber model to about 220% compared with control (* *P *< 0.05). **(b) ***In vitro *assays on cartilage destruction revealed significant induction of free glycosaminoglycans after treatment with Robo3-transfected RASF (late passages; n = 3) compared with mock-transfected cells (** *P *< 0.01). **(c) **Normal skin fibroblasts (n = 2) expressing Robo3 after transfection resulted in the release of significantly more free glycosaminoglycans than control transfected cells (* *P *< 0.05). **(d) **Matrix metalloproteinase (MMP) 1 (n = 3) and MMP3 (n = 4) mRNA expression was quantified by real-time (RT)-PCR, showing significant induction of MMP1 (* *P *< 0.05) and high induction of MMP-3 in mRobo3 transfected RASF (late passages) compared with control. **(e) **MMP1 (n = 3) and MMP3 (n = 4) protein expression from the supernatant released from SF in the GAG assay was quantified via ELISA, showing high induction of MMP-1 and of MMP3 in mRobo3 transfected RASF (late passages) compared to control (* *P *< 0.05).

We then focussed on the significance of the strong upregulation of Robo3 in active RASF. RASF in late passages (P5 and P6) with low Robo3 were transiently transfected using nucleofection with an mRobo3 expression construct [19]. Expression of Robo3 was confirmed by quantitative RT-PCR (data not shown). In Boyden Chamber assays, Robo3-expressing RASF revealed a significant higher migratory ability compared with mock-transfected RASF (Figure 2a).

An *in vitro *test system for quantifying cartilage degradation by SF was used to analyse the effect of Robo3 expression in SF on cartilage destruction (Figure 2b). Here, enhanced degradation of cartilage after Robo3 expression was observed. Additionally, even in normal, dermal fibroblasts derived from skin, expression of Robo3 resulted in a high capacity to degrade cartilage (Figure 2c).

To analyse the molecular details of Robo3 function we determined the expression of MMPs as they are known to be expressed in activated SF and play an important role in cartilage destruction. Re-expression of mRobo3 in RASF in late passages resulted in strong induction of MMP1 and MMP3 mRNA (Figure 2d) whereas MMP13 mRNA stayed unchanged. We could also confirm Induction of MMP1 and MMP3 on protein level by analysing mRobo3 transfected RASF (late passages) supernatant versus control from the GAG assay (Figure 2e).

### Proliferation of RASF and influence of Slit treatment

Slits have been described to have repulsive function in the developing nervous system towards growing axons expressing Robo receptors [6]. However, there are currently no data available that indicates these repellent factors are involved in the regulation of SF activity. We, therefore, analysed the effects of Slit3 (mouse Slit3; mSlit3) on SF of patients with RA.

**Figure 3:**
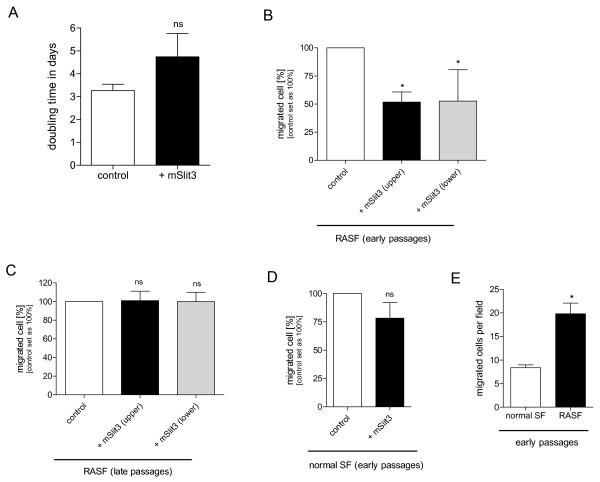
**Functional analysis of Slit3 effects on RASF**. **(a) **Doubling times of rheumatoid arthritis synovial fibroblasts (RASF; n = 2) were obtained from proliferation assays. RASF incubated with mSlit3 showed decreased growth compared to untreated control (ns, not significant). **(b) **Recombinant mouse Slit3 (0.1 μg/ml) decreased migration of RASF in early passages (P3 and P4; n = 4) to about 55% compared with control both in upper and lower chamber (** *P *< 0.01). **(c) **In later passages (P5 and P6), Slit3 treatment has lost its inhibitory effect on migration of RASF compared with control (n = 4). **(d) **Slit3 incubation (0.1 μg/ml) had no effect on migration of normal SF (n = 2) in early passages (P3 and P4) compared with untreated control. **(e) **Relative comparison of migratory ability revealed high induction in RASF compared with normal SF (* *P *< 0.05).

RASF were treated continuously with Slit and proliferation of the cells was followed for five days. Slit3 treatment resulted in a weak but non-significant inhibition of proliferation in RASF (Figure 3a).

### Slit inhibits migration of RASF compared with normal SF

Next, we analysed the influence of Slit3 on migration of RASF. Migration of RASF in early passages (P3 and P4) was strongly inhibited by Slit3 compared with untreated cells (Figure 3b). This effect was evident not only in the presence of Slit3 in the upper chamber with the cells, but also after adding Slit to the lower chamber. This inhibitory effect of Slit3 on RASF completely vanished in later passages (P5 and P6; Figure 3c).

Slit3 treatment had no significant effect on the migration of normal SF (P3 and P4) from healthy donors (Figure 3d).

Relative migration of RASF more than doubled compared with normal SF (both early passages; Figure 3e).

### Robo and Slit expression-changes *in vitro*

**Figure 4:**
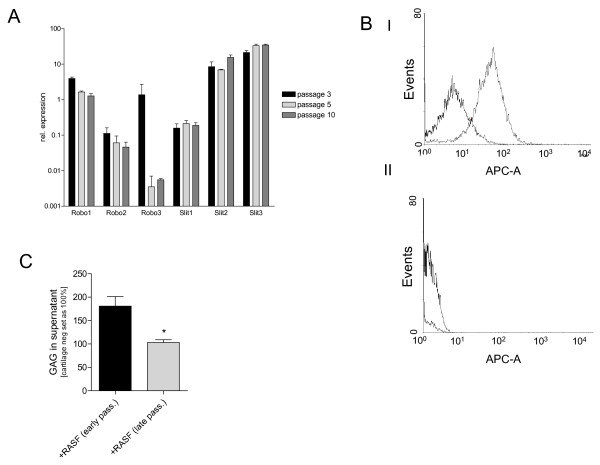
**Robo and Slit expression in RASF in three different passages**. **(a) **Robo1, Robo2, Robo3, Slit1, Slit2, and Slit3 mRNA expression in rheumatoid arthritis synovial fibroblasts (RASF; n = 5) was quantified by real-time (RT)-PCR starting from passage 3 (P3) over passage 5 (P5) to passage 10 (P10; logarithmic scale). Robo1 and Robo2 expression decrease slightly in RASF over the passages. Robo3 mRNA levels dropped dramatically in P5 and P10 compared with P3. No considerable differences in mRNA expression were measured with Slit1, Slit2, and Slit3 over the passages. **(b) **Protein expression of Robo3 in RASF of P3 and P5 was analyzed by FACS using a Robo3 specific antibody. RASF in early passages showed a shift (I) compared with negative control, whereas cells of later passages (II) did not reveal Robo3 expression. **(c) ***In vitro *assays on cartilage destruction revealed significant reduction of free glycosaminoglycans (GAGs) after coculture with RASF of late passages compared with early passages.

As these results hint to phenotypical changes in cell culture, we were interested in the regulation of the expression pattern of Robos and Slits over the passages (P3, P5 and P10) in RASF. In RASF, Robo1 and Robo2 expression slightly decreased from P3 to P10, whereas Robo3 expression showed strong reduction (Figure 4a). Expression of Robo3 in early passages was confirmed by FACS analysis (Figure 4b). Slit1, 2 and 3 expression data showed no significant changes between the passages (Figure 4a). Interestingly, assays on cartilage destruction using RASF in early and late passages revealed reduction of aggressiveness in higher passages which correlates with loss of Robo3 (Figure 4c).

## Discussion

Destruction of the joint in RA is a complex mechanism of deregulation of cartilage homeostasis. In this study, we focused on the repellent factor family Slit and their receptors Robo, which are already known to mediate cell-cell and cell-matrix interactions of migrating cells during embryonic development [8,20]. However, little is known about Slit-Robo regulation in SF, which are known to gain invasive potential especially during RA resulting in cartilage destruction [1,21].

By analyzing the expression pattern of Robos and Slits in SF, we detected strong differences in expression of Robo3 and Slit3 comparing RASF with normal SF.

Upregulation of Robo3 in RASF compared with normal SF may play a role in gaining an aggressive phenotype of SF in RA [5,6]. This hypothesis is supported by our data showing induction of migratory and destructive behavior of SF cells after Robo3 transfection. Interestingly, even dermal fibroblasts gain an aggressive phenotype after expression of Robo3. At least part of this aggressive behavior can be explained by the strong induction of MMP1 and MMP3 after Robo3 expression. Both MMPs are known as markers for RA inflammation [14], which are produced by synovial lining cells and are responsible for matrix degradation. Synovial fluids of RA patients contain about 100-fold higher concentration of MMP3 and increased levels have been found in the sera of patients with RA. MMP1 in the synovial fluid correlates with the degree of synovial inflammation. Upregulation of MMPs after Robo3 transfection might either be regulated via upregulation of pro-inflammatory cytokines, growth factors and matrix molecules [3] or by direct activation via Robo3 signalling.

Slits are known to convey repulsive signals via Robo receptors. Recombinant Slit3 slightly, but not significantly, decreased cell proliferation of RASF and not of normal SF, indicating that Slit3 has only minor influence on cell cycle regulation of human SF.

Several groups could show that Slit can inhibit migration of neuronal cells [1,22] and chemotaxis of leucocytes [23], so we tested the effect of Slit on migration of RASF. Here, we clearly showed that Slit3 inhibits migration of RASF in early passages, whereas these fibroblasts in higher passages and normal SF were not affected. It has been published that Slit2 has a repulsive function towards glioma cells, which is mediated by the Robo1 receptor [1]. So, we speculate that losing repulsive activity towards Slit3 in SF of RA in higher passages (P10) compared with passage 3 might be due to significant reduction of Robo3 expression from passage 3 to 10 and could explain why normal SF are not inhibited in migration by Slit3. This finding further supports the role of Robo3 in RA.

A possible significance of our *in vitro *findings to the pathophysiology of RA is supported by analysis of SNPs within the ROBO3 gene. The SNP marker rs11604758 showed region-wide significant association with susceptibility to RA (*P *corrected for 15 analysed markers = 0.012). This could point to the fact that Robo3 is a potential risk factor for RA. To unravel the genetic contribution to RA risk from *ROBO3 *gene, replication and fine-mapping studies are now required.

## Conclusions

Until today, the treatment of RA is based on a symptomatic therapy with non-steroidal antirheumatic drugs, disease-modifying antirheumatic drugs [24] and on new anti-inflammatory therapies that modulate the immune system and have improved RA treatment [4,25]. However, new strategies try to concentrate on the regulation of activated SF in RA patients (e.g. via dexamethasone) [26], which play an important role in the development of RA. Therefore, the repellent factor family Slit may be a novel therapeutic tool in RA therapy by directly inhibiting SF migration and invasion.

Taken together, our data could show that Slit3 reduces the migratory activity of synovial cells from patients with RA, potentially by repulsion of the cells in analogy to the neuronal system [4,9]. Deregulation of the Robo3 receptor in the SF in RA seems to correlate with aggressiveness of the fibroblasts.

## Abbreviations

BSA: bovine serum albumin; DMEM: Dulbecco's modified Eagle's medium; ELISA: enzyme-linked immunosorbent assay; FCS: fetal calf serum; GAG: glycosaminoglycans; Ig: immunoglobulin; MMP: matrix metalloproteinase; PBS: phosphate-buffered saline; RA: rheumatoid arthritis; RT-PCR: real-time polymerase chain reaction; SF: synovial fibroblasts; SNP: single nucleotide polymorphism.

## Competing interests

The authors declare that they have no competing interests. AB and TS are applying for a patent related to the content of the manuscript.

## Authors' contributions

AD and SK carried out most experiments presented in this study, and were involved in analysis and interpretation of the data. KS was involved in the design of the statistical experiments and in the drafting of the manuscript. TL and JS participated in the interpretation of the data and performed the isolation of primary cells. TS was involved in developing the hypothesis, in designing the study, in analysis and interpretation of the data and in drafting the manuscript. AKB conceived the study and was responsible for its design and coordination, analysis and interpretation of the data and for drafting the manuscript. All authors read and approved the final manuscript.
